# Direct antitumor activity of bevacizumab: an overlooked mechanism?

**DOI:** 10.3389/fphar.2024.1394878

**Published:** 2024-04-23

**Authors:** Zhiyong Wang, Jiaqi Li, Jinjin Guo, Pei Wei

**Affiliations:** Department of Immunology, Zhuhai Campus of Zunyi Medical University, Zhuhai, China

**Keywords:** vascular endothelial growth factor A, bevacizumab, antiangiogenesis, direct antitumor activity, tumor cells

## Introduction

Bevacizumab, a recombinant humanized monoclonal antibody targeting vascular endothelial growth factor A (VEGFA), is pivotal in treating various malignancies (Zondor and Medina, 2004; Pavlidis and Pavlidis, 2013; Kim et al., 2018; Garcia et al., 2020). It primarily neutralizes VEGFA, inhibiting new blood vessel formation necessary for sustained tumor growth and metastasis (Claesson-Welsh and Welsh, 2013; Peach et al., 2018; Apte et al., 2019; Pérez-Gutiérrez and Ferrara, 2023). This anti-angiogenic approach targets the lifeline of the tumor—its vascular systems, focusing on endothelial cells. However, it is widely acknowledged that the role of VEGFA in cancer extends beyond angiogenesis. VEGFA influences the function of stromal and immune cells within the tumor microenvironment and directly modulates the functionality of tumor cells (Goel and Mercurio, 2013; Pérez-Gutiérrez and Ferrara, 2023), particularly in promoting their survival and proliferation. Therefore, it leads to an intriguing paradox in the therapeutic role of bevacizumab. Many tumor cells express high levels of VEGFA and its receptors/co-receptors (Lalla et al., 2003; Donnem et al., 2009; Capp et al., 2010; Goel and Mercurio, 2013; Nordby et al., 2015; Morimoto et al., 2019); thus, bevacizumab can theoretically exert direct cytotoxic effects on these cells by blocking VEGFA. However, tumor cells have evaded the direct cytotoxic effect of bevacizumab, and the underlying reasons remain unexplained.

For years, bevacizumab’s purported lack of direct inhibitory effects on tumor cells was primarily based on early *in vitro* experiments, which failed to demonstrate significant direct cytotoxic effects (Naumov et al., 2009; Hasan et al., 2011; Hein and Graver, 2013; Mesti et al., 2014). However, the tide of evidence is shifting. Increasingly, studies suggest that bevacizumab does exert direct cytotoxic effects on encountered tumor cells (Miranda-Goncalves et al., 2017; Zhao et al., 2018; Suo et al., 2020; Alonso-Diez et al., 2021; Fan et al., 2022; Wei et al., 2023), with even bevacizumab-induced cell death observed in certain transplant tumor models (Ramezani et al., 2017; Zhao et al., 2018; Ramezani et al., 2019; Xiang et al., 2022). These emerging findings challenge the notion that bevacizumab lacks direct anti-tumor activity, prompting a need for rigorous scrutiny and scientific exploration.

In this opinion article, we re-examined bevacizumab’s anti-tumor mechanism. A comprehensive understanding of its therapeutic potential necessitates considering its direct cytotoxic effects on tumor cells alongside its anti-angiogenic action. Our goal is to emphasize bevacizumab’s direct anti-tumor action and elucidate why its historical oversight of direct cytotoxic effects on tumor cells have historically been overlooked. Incorporating bevacizumab’s direct cytotoxic effects on tumor cells into its mechanism of action represents not only an academic advancement, but also carries significant implications for its clinical application.

## Traditional mechanism of action of bevacizumab and its challenges

Bevacizumab’s antitumor activity traditionally stems from its suppression of angiogenesis. This mechanism relies on the premise that tumor growth and metastasis necessitate new blood vessel formation to ensure oxygen and nutrient supply (Sherwood et al., 1971; Bergers and Benjamin, 2003). Pro-angiogenic factors perpetuate tumor blood vessel formation, with VEGFA being the most critical (Claesson-Welsh and Welsh, 2013; Apte et al., 2019). VEGFA binds to VEGFRs on endothelial cells, triggering downstream signaling pathways that promote endothelial cell proliferation, migration, and new blood vessel formation (Peach et al., 2018; Pérez-Gutiérrez and Ferrara, 2023). Bevacizumab binds to circulating VEGFA, hindering its interaction with VEGFRs on endothelial cell surfaces, thereby impeding crucial steps in tumor blood vessel formation. This inhibition not only halts new blood vessel formation but also impairs the functionality of existing tumor blood vessels. Consequently, by inhibiting this process, bevacizumab effectively starves the tumor of vital nutrition and oxygen, thus stunting its growth. Moreover, anti-VEGFA therapy has a significant impact on the tumor microenvironment (Finley and Popel, 2013; Tu et al., 2023), which has promoted the development of combined treatment strategies of anti-VEGFA with other tumor immunotherapies such as immune checkpoint inhibitors (Hack et al., 2020; Chambers et al., 2021; Zheng et al., 2022; Tu et al., 2023). New blood vessels typically have structural abnormalities and have increased permeability, crucial for tumor spread and metastasis. Bevacizumab indirectly suppresses tumor cells’ invasive and metastatic potential by improving the quality of these vessels and reducing their number. Additionally, it recalibrates abnormal tumor blood vessels, causing a brief “normalization” period (Goel et al., 2011; Chen et al., 2016; Zhang et al., 2021; Li et al., 2022). During this window, previously tortuous and highly permeable tumor blood vessels become more organized and leak less, improving tissue perfusion and drug delivery. This phenomenon offers another therapeutic angle: enhancing the efficacy of combined chemotherapy drugs and radiotherapy (Becker et al., 2015; Kulinich et al., 2021; Socinski et al., 2021; Hardesty et al., 2022).

While bevacizumab is effective in treating tumors, certain clinical challenges have highlighted its complex mechanism of action. For instance, despite high expression levels of VEGFA and its receptors in patients, responses to bevacizumab vary significantly. Some patients respond well, while other patients exhibit resistance (Giantonio et al., 2007; Haunschild and Tewari, 2020; Dai et al., 2022). VEGFA-independent angiogenesis is considered the primary reason for bevacizumab resistance; however, numerous attempts to inhibit VEGFA-independent angiogenesis have failed to reverse this resistance. These issues pose serious challenges to traditional anti-angiogenic mechanisms involving bevacizumab. As such, research should also explore its effects in addition to its anti-angiogenic properties. With research progress, studies have focused on the direct cytotoxic effects of bevacizumab on tumor cells.

## Beyond angiogenesis: direct cytotoxic effects of bevacizumab on tumor cells

The *in vivo* tumor microenvironment complexity has led to a reliance on *in vitro* cell cytotoxicity assays to demonstrate the direct antitumor efficacy of drugs. Early *in vitro* studies significantly influenced our comprehension of bevacizumab’s antitumor activity, predominantly indicating that its direct inhibition of tumor cells might be inconsequential (Naumov et al., 2009; Hasan et al., 2011; Hein and Graver, 2013; Mesti et al., 2014). This prompts the question: why do tumor cells, bathed in high VEGFA concentrations and theoretically susceptible to bevacizumab, seemingly resist its direct cytotoxic effects? Despite acknowledging the multifaceted role of the VEGFA signaling axis in tumors, extending beyond angiogenesis regulation alone, this puzzle persists. As our understanding of bevacizumab’s pharmacological characteristics deepens, numerous findings challenge longstanding beliefs. This is evident in bevacizumab’s capacity to induce direct cytotoxic effects in various tumor cells expressing high VEGFA levels (Miranda-Goncalves et al., 2017; Zhao et al., 2018; Suo et al., 2020; Alonso-Diez et al., 2021; Fan et al., 2022; Wei et al., 2023). At the molecular level, studies proposed mechanisms through which bevacizumab induces tumor cell death. One mechanism involves blocking VEGFA-mediated survival pathways, increasing apoptosis. Another potential pathway is the alteration of intracellular signaling cascades crucial for tumor cell homeostasis. Bevacizumab may disrupt these pathways’ stability, resulting in direct cytotoxic effects. *In vivo* models further support the direct cytotoxic hypothesis, showing bevacizumab’s ability to induce cell death within tumor masses in xenograft models, evident from increased apoptosis and morphological changes in tumor cells (Ramezani et al., 2017; Zhao et al., 2018; Ramezani et al., 2019; Xiang et al., 2022). Traditional metrics like tumor volume and microvascular density may not fully capture these changes, suggesting additional mechanisms at play and reinforcing the concept of a multipronged attack on cancer cells.

Table 1 summarizes the inconsistent results of studies on the direct cytotoxic effects of bevacizumab on various VEGFA-expressing tumor cells. For instance, studies on the same tumor cell line A549 at similar concentration ranges (0.0001–0.2 mg/L) and identical time points (24–72 h) have shown conflicting results; that is, some studies have reported that bevacizumab elicits no direct cytotoxic effects (Naumov et al., 2009; Liu et al., 2022), while other studies have revealed significant findings (Wang et al., 2015; Xiao et al., 2016; Wei et al., 2023). On examining these literatures, it appears that studies confirming bevacizumab’s direct cytotoxic effects often employ a variety of methods to assess its direct cytotoxicity. Thus, the choice of evaluation method might have been a crucial factor previously overlooked in recognizing the direct cytotoxic effects of bevacizumab.

**TABLE 1:** Summary of the direct cytotoxic effect of bevacizumab on tumor cells.

Cancer types	Cell lines	Dosage/Time	Direct cytotoxicity (Yes/No)	References
Non-small cell lung cancer	A549, H1650, H3255, Calu6, H1975, HCC827	0.0001–0.01 mg/mL/72 h	No	Naumov et al. (2009)
Colorectal cancer	MIP101, RKO, HCT116	0.01–0.05 mg/mL/96 h	No	Hasan et al. (2011)
Glioma	C6	2.5–10 mg/mL/72 h	No	Xu et al. (2014)
Glioma	U87	0.00001–0.25 mg/mL/24 or 72 h	No	Mesti et al. (2014)
Glioma	mutant IDH1-U87	0.1–1 mg/mL/72 h	No	Mesti et al. (2018)
Breast Cancer	MDA-MB-231	0.05 mg/mL/24 h	No	El-Hajjar et al. (2019)
Uveal melanoma	MEL-270, OMM-2.5	0.25 mg/mL/72 h	No	Tura et al. (2019)
Oral squamous cell carcinoma	SAS, HSC-2	0.001–0.1 mg/mL/24 h	No	Itashiki et al. (2021)
Non-small cell lung cancer	A549, H1299	0.005–0.2 mg/mL/24 h	No	Liu et al. (2022)
Hepatocellular carcinoma	HepG2	0.25 mg/mL/24, 48 or 72 h	No	Taha et al. (2022)
Breast Cancer	MCF7, NH27, MDA-MB-231, SKBR3	0.00001–0.1 mg/mL/144 h	MCF7, NH27 No	Emlet et al. (2007)
			MDA-MB-231, SKBR3≥0.001 mg/mL Yes	
Melanoma	VMM18, DM6, DM18, DM93, DM122, VMM39	0.05 mg/mL/48 h	DM93, DM122, VMM39 No	Molhoek et al. (2008)
			VMM18, DM6, DM18 Yes	
Melanoma	B16F10	0.23–4.6 mg/mL/24, 48, or 72 h	Others No 4.6 mg/mL/72 h Yes	Filali et al. (2012)
Uveal melanoma	MEL285, OMM2.3		≥2.3 mg/mL Yes	
Hepatocellular carcinoma	SMMC-7721	0.001–0.02 mg/mL/48 or 72 h	Others No 0.02 mg/mL Yes	Wang et al. (2021)
Prostate cancer	C4-2B	0.1 mg/mL/24, 48, or 72 h	≥48 h Yes	Yang et al. (2012)
Retinoblastoma	SNUOT-Rb1	0.1–10 mg/mL/48 h	10 mg/mL Yes	Heo et al. (2012)
Ovarian cancer	HO-8910, HO-8910PM	0.01 mg/mL/24, 48 or 72 h	≥48 h Yes	Zhang and Zou (2015)
Non-small cell lung cancer	A549	0.15–3.75 mg/mL/24 or 72 h	≥0.15 mg/mL/48 h Yes ≥0.75 mg/mL/24 h Yes	Wang et al. (2015)
Non-small cell lung cancer	A549	0.0001–0.005 mg/mL/24 h	≥0.001 mg/mL Yes	Xiao et al. (2016)
Glioma	SW1088, U251, A172, U87, SNB-19, GAMG, SW1783	0.5–3 mg/mL/72 h	≥0.5 mg/mL Yes	Miranda-Goncalves et al. (2017)
Glioma	U87	0.5–32 mg/mL/24 or 48 h	≥0.5 mg/mL/48 h Yes ≥1 mg/mL/24 h Yes	Huang et al. (2018)
Colorectal cancer	HT29	5–30 mg/mL/36 h	≥0.5 mg/mL Yes	Zhao et al. (2018)
Ovarian cancer	A2780	0.001–0.02 mg/mL/96 h	≥0.005 mg/mL Yes	Zhang et al. (2019)
Colorectal cancer	SW480, SW620	0.001–0.003 mg/mL/48 h	≥0.003 mg/mL Yes	Suo et al. (2020)
Breast Cancer	IPC-366, SUM149	0.2–0.8 mg/mL/24, 48, or 72 h	0.4 mg/mL/48 or 72 h Yes 0.8 mg/mL Yes	Alonso-Diez et al. (2021)
Ovarian cancer	SKOV3	0.5–3 mg/mL/48 or 72 (h)	≥1 mg/mL/72 h Yes	Fan et al. (2022)
Glioma	U251	0.2 mg/mL/48	Yes	Wei et al. (2023)
Non-small cell lung cancer	A549			
Colorectal cancer	HT29			
Breast Cancer	MDA-MB-231			

## Limitations of *in vitro* experimental methodologies

When assessing bevacizumab’s direct cytotoxic effects, it is crucial to recognize the limitations inherent in traditional *in vitro* evaluations. Standard anti-tumor assays expose tumor cells to bevacizumab for 24–72 h, relying on end-point measurements for direct cytotoxic effects determination. These methods often focus on a single biomarker, such as cell membrane integrity or metabolic activity, disregarding the multifactorial and multi-stage nature of cell death and survival, leading to oversimplified conclusions about cellular states (Adan et al., 2016; Parboosing et al., 2016; Halim, 2020).

For example, colorimetric assays like 3-(4,5-Dimethylthiazol-2-yl)-2,5- diphenyl tetrazolium bromide (MTT) and water soluble tetrazolium (WST) assays, widely used for quantifying viable cells *in vitro* (Berridge et al., 2005; Lutter et al., 2017; Präbst et al., 2017; Stockert et al., 2018), are common for assessing bevacizumab’s direct cytotoxic effects (Naumov et al., 2009; Hasan et al., 2011; Hein and Graver, 2013; Mesti et al., 2014). These assays operate on the principle of converting soluble tetrazolium salts into insoluble formazan by living cells, measurable through absorbance changes upon dissolution in a solvent. However, research suggests that tetrazolium salt reduction is significantly influenced by various factors, notably changes in cellular metabolism—a process that bevacizumab has been shown to induce through metabolic reprogramming in tumor cells (Zulato et al., 2012; Miranda-Goncalves et al., 2017; Yue and Lu, 2023). Thus, in our previous research, we identified the use of tetrazolium-based colorimetric assays as a methodological oversight in directly assessing bevacizumab’s direct cytotoxic effects on tumor cells (Wei et al., 2023). Specifically, MTT or WTS assays have shown that 0.2 mg/L bevacizumab does not significantly exhibit direct cytotoxic effects on tumor cells. Conversely, precise counting has revealed that bevacizumab inhibits tumor cell growth by 40%–47%. This discrepancy occurs because bevacizumab enhances mitochondrial metabolism in tumor cells. As a result, it increases the production of succinate dehydrogenase that consequently reduces more tetrazolium salts to formazan, indicating an increased number of living cells.

Furthermore, the end-point nature of such analyses obscures the dynamic morphological and biochemical changes that occur during exposure to bevacizumab. Given the tumor cells’ heterogeneity, it is plausible that while some succumb to bevacizumab’s effects and undergo cell death, others adapt and proliferate, possibly through metabolic shifts or alterations in their secretory profiles. Consequently, the ultimate measurement might reflect an apparent equilibrium between cell death and compensatory proliferation, masking bevacizumab’s true direct cytotoxic effects. For example, El-Hajjar et al. (2019) found that the number of tumor cells doubled after 24 h of treatment with 0.05 mg/mL bevacizumab compared with that of the control group. This increase could be a result of compensatory proliferation exceeding the number of dead cells.

In summary, these methodological subtleties could inadvertently mask the direct cytotoxic effects, presenting an incomplete view of bevacizumab’s capabilities. Consequently, we propose a redefined framework to comprehend bevacizumab’s role in cancer therapy, one that incorporates both its direct anti-tumor and anti-angiogenic properties.

## Redefining the antitumor mechanism of bevacizumab

**FIGURE 1:**
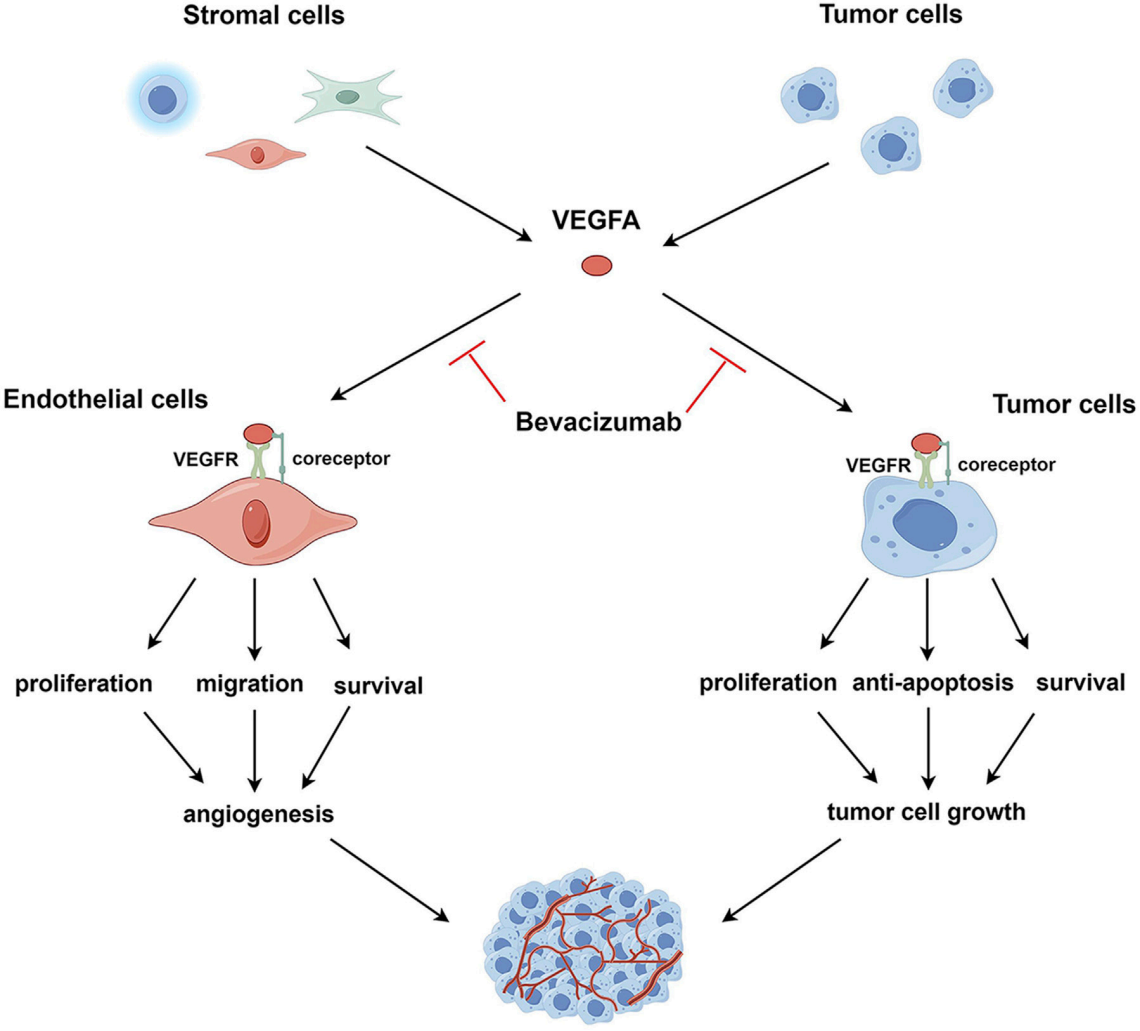
VEGFA signaling in endothelial and tumor cells. VEGFA, secreted by tumor and stromal cells, such as endothelial cells, immune cells, and fibroblasts, binds to VEGFR and co-receptors on the surface of endothelial or tumor cells, activating intracellular VEGFA signaling pathways. In endothelial cells, activated VEGFA signaling promotes cell survival, proliferation, and migration, leading to the induction of tumor angiogenesis. In tumor cells, the activation of the VEGFA pathway enhances cell survival, proliferation, and resistance to apoptosis, facilitating tumor cell growth. VEGFA targets both endothelial and tumor cells; correspondingly, bevacizumab exerts anti-angiogenic and direct antitumor effects by neutralizing VEGFA.

Here, as depicted in Figure 1, we propose an expanded mechanistic framework that involves the direct cytotoxic effects of bevacizumab on tumor cells and thus emphasizes its ability to target both the tumor vasculature and the tumor itself. Specifically, bevacizumab weakens angiogenesis by inhibiting VEGFA signaling; as a consequence, tumors are deprived of critical nutrients and oxygen. This antibody also directly binds to VEGFA-expressing tumor cells, thereby triggering apoptosis pathways and inhibiting proliferation and survival signaling.

Recognizing this composite mechanism of action—targeting angiogenesis and tumor cells alike—has profound implications for oncological treatment strategies. Traditionally, bevacizumab’s impact was understood mainly in terms of its effect on the tumor’s blood supply through VEGFA inhibition, but this does not fully explain the lack of sensitivity observed in certain highly vascularized tumors among some patients. If bevacizumab exerts direct cytotoxic effects on VEGFA-expressing tumor cells, individual variances in VEGFA expression levels might be a key determinant in the variability of therapeutic responses. Furthermore, when devising treatment regimens, clinicians might tailor therapies based on the VEGFA expression levels in a patient’s tumor cells. For instance, identifying markers indicative of a direct tumor cell response will become as critical as those suggesting angiogenesis when predicting treatment outcomes. Additionally, understanding the temporal patterns of tumor response and resistance to bevacizumab could offer avenues to circumvent the emergence of resistant cancer cell populations. Once bevacizumab suppresses VEGFA within the tumor microenvironment, it affects the tumor’s ability to access oxygen and nutrients, not only through reduced vasculature but potentially by exerting direct survival pressures on the tumor cells. The cells must then adapt to these new conditions to sustain growth, a process that may involve activating alternative growth factor pathways, enhancing intracellular signaling to compensate for growth factor deprivation, or modulating apoptosis mechanisms to evade death signals. Elucidating these critical aspects will aid in preventing or overcoming resistance to bevacizumab.

Finally, incorporating bevacizumab’s direct cytotoxic effect enriches our understanding of its mechanism, fostering a comprehensive view of the tumor microenvironment—a complex ecosystem encompassing tumor cells, vessels, immune cells, and diverse cellular types and molecular signals. Following bevacizumab treatment, the tumor microenvironment may undergo significant changes such as cytokine and growth factor profile shifts, immune cell infiltration alterations, and extracellular matrix component modifications. These multifactorial effects may arise not only from anti-angiogenic actions but also from bevacizumab’s direct pressure on tumor cell survival.

In summary, under the new mechanistic framework, the therapeutic effects of bevacizumab are possibly attributed to a combination of its anti-angiogenic and direct cytotoxic effects. As such, drug administration protocols, therapeutic combinations, and predictive biomarkers should be reassessed to optimize the therapeutic outcomes of bevacizumab.

## Further research is required to validate the direct cytotoxic effects of bevacizumab

A multifaceted experimental approach should be used to address the gaps in our understanding of the cytotoxic effects of bevacizumab on tumor cells. This strategy should initially commence with *in vitro* experiments to verify the direct cytotoxic effects of bevacizumab on cancer cells. With limitations inherent to endpoint assays, advanced imaging technologies should be adopted to monitor cellular responses in real time. In this way, cell death and potential compensatory proliferation, which can obscure the interpretation of the efficacy of bevacizumab, can be detected. Fluorescent markers specifically targeting cell death indicators, such as caspases, can be used to intricately analyze drug-induced cell death; routine medium replacement can prevent the confounding effects of proliferative cytokines that accumulate in a culture environment. Moreover, appropriate control cell lines should be chosen to elucidate the specificity of the cytotoxic effects of bevacizumab. Cells devoid of VEGFA expression and its receptors are key controls to definitively attribute the observed cytotoxicity to the disruption of the VEGFA signaling pathway. Furthermore, the effect of bevacizumab on normal cells expressing VEGFA and its receptors should be assessed to predict potential adverse effects accurately. Additionally, current research findings indicate that the direct cytotoxic effects of bevacizumab are not cell type-specific, as it exhibits significant cytotoxicity across 26 cell types from up to 8 different tumor classes. These findings also suggest that the direct cytotoxic effects of bevacizumab generally increases with concentration and time. However, it has been noted that these studies do not clearly define the relative expression levels of VEGFA and its receptors/co-receptors in different cells, nor do they test each cell’s sensitivity to a unit concentration of VEGFA. Therefore, it is imperative to re-evaluate the direct cytotoxic effects of bevacizumab on various tumor cells, upon clarifying the relative expression levels of VEGFA and its receptors/co-receptors, as well as the sensitivity of different cells to unit concentrations of VEGFA. This will aid in further elucidating the mechanisms through which bevacizumab exerts its direct cytotoxic effects. Subsequently, once the specific cytotoxic effects of bevacizumab against tumor cells are validated, the potential development of tumor cell resistance to this direct action should be explored. Such insights remarkably help reveal resistance mechanisms and reaffirm the potency of bevacizumab in inducing cell death. Furthermore, considering the standard incorporation of bevacizumab with chemotherapeutic agents in clinical settings, researchers should assess the *in vitro* synergistic cytotoxic effects of bevacizumab and various chemotherapeutics by investigating how tumor cells may adapt to resist this combination therapy. Thus, valuable guidance for clinical applications can be provided. Finally, *in vitro* findings should be translated into *in vivo* contexts to verify the observed mechanisms and effects. However, sophisticated experimental designs are necessary to distinguish between the direct cytotoxic effects of bevacizumab and those mediated indirectly through immune modulation. They may involve comparing outcomes between immunocompromised and immunocompetent animal models to elucidate the contribution of immune-mediated effects to the overall antitumor activity of bevacizumab.

## Challenges and limitations in the clinical translation of bevacizumab’s direct cytotoxic effects

Although the direct cytotoxic effects of bevacizumab on tumor cells *in vitro* show potential for cancer therapy, translating these findings into clinical practice is impeded by limitations. First, tumors have inherent heterogeneity. The genetic and phenotypic diversity within and among tumors can considerably influence the efficacy of bevacizumab; as such, predicting responses based solely on *in vitro* data becomes difficult. Because of this variability, personalized approaches should be implemented to select patients who will likely benefit from bevacizumab treatment, emphasizing the importance of identifying response-predictive biomarkers. Second, the optimal concentration of bevacizumab that reflects the effective *in vitro* doses cannot be easily determined because of factors such as physiological barriers to drug delivery, systemic toxicity, and varying pharmacokinetics across patients. For this reason, dosing regimens should be cautiously optimized to maximize efficacy while minimizing adverse effects; thus, extensive clinical trials should be performed to establish safe yet effective dosage guidelines. Furthermore, the direct cytotoxic effects observed in isolated cell environments do not account for the dynamic interplay with the patient’s immune system. *In vivo*, the interaction of bevacizumab with immune mechanisms influences its effectiveness, but such interaction complicates the direct translation of its cytotoxic effects. Therefore, as a multidirectional avenue for future research, understanding how bevacizumab modulates immune system responses against tumors is crucial for achieving its full therapeutic potential. Moreover, the combination of bevacizumab with chemotherapeutic or targeted agents is a routine clinical strategy; therefore, incorporating the direct cytotoxic effects into the mechanism of action of bevacizumab undoubtedly further expands its potential for combination therapy. This potential not only lies in the “vascular normalization” induced by bevacizumab, making it easier for drugs to enter the tumor, but also in the added direct cytotoxic effects on tumor cells when used in conjunction with chemotherapeutic or targeted agents. Considering the important role of VEGFA in chemotherapy or targeted drug resistance, especially in regulating cancer stem cells (Goel and Mercurio, 2013; Mercurio, 2019), the combination of bevacizumab is also very attractive for preventing the development of drug resistance. However, just as a coin has two sides, if drug resistance occurs during the combination treatment, it also means the formation of more complex drug resistance mechanisms, making it more challenging to overcome resistance. Ultimately, bevacizumab should be integrated into cancer treatment regimens to improve patient outcomes, including extending survival times and enhancing the quality of life. However, the straightforward relationship between *in vitro* cytotoxicity and patient-centric endpoints is complicated by biological, environmental, and treatment-related factors. Therefore, a holistic approach should be implemented in clinical trials to evaluate not only the efficacy of bevacizumab but also its effect on overall patient wellbeing.

Although transitioning from the controlled environment of a laboratory to the complex realities of patient treatment has numerous challenges, this process is necessary to achieve the potential of bevacizumab for cancer therapy. By recognizing these obstacles and investing in interdisciplinary collaborative research efforts, we can narrow the gap between *in vitro* findings and clinical applications; thus, we can advance the development of targeted and effective cancer treatments.

## Conclusion

This opinion article revisits bevacizumab’s antitumor activity mechanisms, underscoring the necessity to consider its direct cytotoxic effects on tumor cells alongside traditional angiogenesis inhibition views. Moreover, it also examines endpoint measurement technique limitations in evaluating bevacizumab’s cytotoxic impact, highlighting methodological oversights in previous studies that ignored its direct cytotoxic efficacy. Expanding our understanding of bevacizumab’s mechanism not only enriches comprehension of its therapeutic modality but also offers new perspectives for devising and refining cancer treatment strategies. However, fully grasping bevacizumab’s anticancer potential and optimal clinical application requires further investigation. Future research should focus on deeper exploration of bevacizumab’s direct mechanisms and translating these insights into clinical practices to enhance therapeutic outcomes for cancer patients.
